# High HIV Positivity Rates Following Large-Scale HIV Self-Testing Implementation in Zimbabwe, 2018–2020

**DOI:** 10.3389/fpubh.2021.606376

**Published:** 2021-03-23

**Authors:** Auxilia Muchedzi, Mulamuli Mpofu, Fungai H. Mudzengerere, Moses Bateganya, Tarirai Mavimba, Hind Satti, Rumbidzai Dhliwayo, Tinashe Zulu, Talent Tapera, Tendai Samushonga, Tendai Nyagura, Getrude Ncube, Taurayi A. Tafuma

**Affiliations:** ^1^FHI 360, Harare, Zimbabwe; ^2^FHI 360, Gaborone, Botswana; ^3^FHI 360, Durham, NC, United States; ^4^United States Agency for International Development (USAID), Harare, Zimbabwe; ^5^Ministry of Health and Child Care, Harare, Zimbabwe

**Keywords:** Zimbabwe, HIV, self-testing, Sub-Saharan Africa, HIV testing

## Abstract

**Introduction:** HIV self-testing (HIV-ST) is an innovative strategy to increase HIV case identification. This analysis shares the outcomes of HIV-ST implementation within the Zimbabwe HIV Care and Treatment (ZHCT) project for the period October 2018–March, 2020.

**Materials and Methods:** We extracted HIV-ST data for the period October 2018 to March 2020 from the project database and assessed (1) the proportion of reactive HIV-ST results; (2) the concordance between reactive HIV-ST results against rapid confirmatory HIV tests using Determine™ and Chembio™ in parallel; and (3) the monthly contribution of HIV-ST to total HIV positive individuals identified within project. The Chi-square test was used to assess for statistical differences in HIV positivity between age groups, by sex and district; as well as the difference in HIV positivity between the HIV-ST and index and mobile testing strategies.

**Findings:** Between October 2018 and March 2020, the ZHCT project distributed 11,983 HIV-ST kits; 11,924 (99.8%) were used and 2,616 (21.9%) were reactive. Of the reactive tests, 2,610 (99.8%) were confirmed HIV positive giving a final positivity rate of 21.9%, and a concordance rate of 99.8% between the HIV-ST results and the confirmatory tests. Proportion of reactive results differed by age-groups (*p* < 0.001); with the 35–49 years having the highest positivity rate of 25.5%. The contribution of HIV-ST to total new positives increased from 10% in October 2018 to 80% at the end of March 2020 (*p* < 0.001). Positivity rates from HIV-ST were significantly different by age-groups, sex and district (*p* = 0.04). Additionally, index and mobile testing had a higher positivity rate compared to HIV-ST (*p* < 0.001).

**Conclusion:** The ZHCT project has successfully scaled up HIV self-testing which contributed significantly to HIV case finding. Countries should consider using the lessons to scale-up the intervention which will contribute in reaching under-served and undiagnosed populations.

## Introduction

In 2016, Zimbabwe had an estimated 1.2 million PLHIV, and an adult prevalence of 14.1% (1), a huge decline from 27.2% in 1998 (2). During the same period, incidence also declined from 2.6% to 0.47%. Pivotal to the national success was the decentralization and scale-up of HIV testing and treatment services from 337 central, district, and primary hospitals in 2009 to 1,552 primary health facilities by 2016 (3). As of 2019, an estimated 76.8% PLHIV in Zimbabwe knew their HIV positive status of which 88.4% were on ART (4). These are positive milestones toward the achievement of the Joint United Nations Program on HIV and AIDS (UNAIDS) 95-95-95 Goals (5). While significant progress has been made on the 1st 95 which focuses on case finding, gaps still exist in reaching some under-served populations.

HIV self-testing (HIV-ST) is an innovative and high-impact strategy to increase HIV-case identification (6). It offers greater convenience and privacy, and has the potential to increase the proportion of the population who test regularly (6). HIV-ST has been found to be highly acceptable to young people in several African countries as it empowers them to choose the location and timing of the test and control disclosure around their results (7, 8). In pilot studies conducted in Zimbabwe, Zambia and Malawi, the highest proportions of first-time testers through HIV-ST were in young men and women in the 16–24 year age group, and men older than 50 years of age (9), population groups known to be lagging behind in the 1st 95. Unsupervised HIV-ST has also been found to be feasible in rural Africa and is non-inferior to provider-supervised HIV-ST (10), but demand has been highly price sensitive (11).

In this study conducted in Zimbabwe, HIV-ST kits were introduced within an established project, the 6-year PEPFAR/USAID funded Zimbabwe HIV Care and Treatment (ZHCT) led by FHI 360, which was implementing HIV testing through (i) mobile clinics in targeted hotspots and (ii) index case testing at homes since 2016. HIV-ST was offered to sexual contacts of newly tested HIV positive individuals referred as index cases at home, and also distributed at hotspots during targeted mobile testing. In this analysis, we assessed the proportion of self-testers screened and confirmed HIV-positive, following a multi-year, large scale implementation of HIV-ST. Results from this real-world approach to scaling up HIV-ST will help demonstrate the approaches of implementing HIV-ST in order to realize its utility in case finding.

## Materials and Methods

Using programmatic data, we evaluated HIV-ST uptake and HIV-positivity rates within the ZHCT project by month from October 2018 to March 2020.

### Implementation Approach of HIV-ST Within ZHCT

Through the ZHCT project, HIV-ST was implemented in eight Districts namely Mwenezi, Kwekwe, Makoni, Gutu and Gokwe South, Zaka, Chivi and Mutasa. The project deliberately scaled up HIV-ST in a phased rollout starting with three districts (Mwenezi, Makoni, and Kwekwe) as a pilot in October 2018. Two districts were added in March 2019 (Gokwe South and Gutu) before further expansion to all eight districts in October 2019. Kwekwe and Makoni Districts are partly urban and partly rural while the rest of the districts are rural. Two of the districts, Kwekwe (HIV prevalence = 14.42%) and Chivi (14.19%) have an HIV prevalence which is higher than the national average of 14.1%. Mutasa District has the lowest prevalence of 9.46% amongst all the eight districts.

After the first 5 months, further rollout of HIV-ST was informed by early lessons from the three-district pilot. For example, because more reactive results were among males, subsequent distribution targeted men; and because most people (75%) who received tests kits during the pilot needed assistance, we offered support more during subsequent rollout. Finally because preliminary data showed that HIV-ST reduced the number of HIV tests conducted by nurse testers thereby reducing workload, we used this information to promote HIV-ST and get further buy in from staff. Prior to introduction of HIV-ST, the ZHCT project had been implementing index testing in the community and targeted mobile testing since 2016. Clients reached were tested by project nurse testers (who are qualified general nurses) using rapid HIV Type 1 antibody testing in line with the national HIV testing algorithm. To reduce the number of clients directly tested by nurse testers, the project introduced screening through HIV-ST using the OraQuick test kit (OraQuick ADVANCE® Rapid HIV-1/2 Antibody Test, OraSure Technologies, Inc.) ahead of HIV Type 1 antibody testing within its index and mobile testing strategies.

Ahead of index testing, HIV-ST was provided at household level or at targeted hotspots to the sexual contacts of index cases that were aged 16 years and above who were followed up by community outreach workers. Index cases were clients who would have recently returned a confirmed positive HIV test and identified from HIV testing registers at health facilities within the supported districts; and any clients who newly tested HIV positive through index and mobile testing at home or targeted hotspots within the ZHCT project. All index cases whether identified at the facility or community were listed in the index case testing (ICT) register which were based at facility level. Outreach workers [community-based expert clients and described here (12)] extracted names of index cases from the facility ICT register, then elicited contacts before following them up in the community where they distributed the HIV-ST kits.

The outreach workers prepared contacts of index cases by making appointments with those who agreed to be tested for HIV, with HIV-ST being offered as the initial test. The date, time, and place for meeting for HIV-ST distribution and testing were recorded in the appointment diaries. The outreach workers, where requested, assisted clients to conduct the HIV-ST and/or interpreted the result. They also collected information on prior HIV testing, demonstrated how the test is done and how the results are interpreted. Clients who did not require direct assistance were given written instructions as well as a video which they could play for guidance for those with compatible phones. After the test, the community outreach workers obtained the results from the clients which they reported daily to the nurse tester responsible for that community. The outreach worker will then contacted those who would have returned a reactive result and book an appointments for a confirmatory test which was done at the household level by the nurse tester. The HIV confirmatory tests are conducted in accordance with national guidelines (13), using Determine™ (Alere Determine™ HIV−1/2 Ag/Ab Combo) and Chembio™ (Chembio HIV 1/2 STAT-PAK®) rapid HIV Type 1 (HIV-1) p24 antigen (Ag) and HIV Type 1 antibody test kits in parallel. Those who tested positive on Determine™ and Chembio™ were recorded as HIV positive and referred for ART. Aggregate data on positivity and contribution to positives were reviewed weekly as part of program monitoring and lessons shared across sites.

HIV-ST is also implemented during mobile testing at targeted hot spots, mainly farming and mining communities, and long-distance truck-ins where target populations were famers, artisanal miners, female sex workers and truck drivers. Outreach workers made available HIV-ST kits to individuals who they would have considered at high risk of HIV, based on a screening tool that assessed prior HIV testing and results, assessed clinical symptoms (e.g., TB, STI), behaviors or social practices and those who belonged to specific social networks. Apart from those determined as eligible using the screening tool, HIV-ST was also made available on request to other adults found within hotspots. Outreach workers, who are expert patients used their community intelligence and their knowledge on HIV clinical symptoms to initiate conversation with targeted population groups. Targeted and consenting individuals were given HIV-ST kits, instructed how to test and those with a reactive result were confirmed by a project nurse tester as described above. However, the nurse tester was always part of the team that conducted outreaches at hotspots for testing.

Children were not offered HIV-ST but were offered HIV Type 1 antibody test conducted by nurse testers. Additionally, adults who decline HIV-ST were directly tested by nurse testers as part of index or targeted mobile testing. Service providers in this project received training on the WHO Five Cs of HIV testing to assure confidentiality (Consent, confidentiality, counseling, correct test results and connections to care). Consent was sought at the time of booking an appointment, with the client given the opportunity to suggest the best time, day, and place for HIV testing. Privacy was ensured by allowing them to choose alternative private and suitable best places for the testing.

### Data Collection

During delivery of services, outreach workers compiled data on HIV-ST kits distributed and recorded all clients in the HIV-ST register. Following confirmatory testing for clients, the information was captured in the community HTS register. The district Monitoring and Evaluation Officer (MEO) aggregated data from the HIV-ST registers every month and entered it into the ZHCT project database, a DHIS 2 aggregate-based health information system.

Data for this analysis was extracted from the project DHIS 2 aggregate system. The variables extracted were number of HIV-ST kits distributed, HIV-ST results returned, HIV-ST results reactive, and HIV-ST reactive and confirmed by nurse tester. We also extracted HIV testing data for children who were not eligible for HIV-ST and data for adults who declined HIV-ST but received testing from nurse testers through index or targeted mobile testing. The data extracted was disaggregated by age and sex, and it was sent to the district MEO for validation.

### Data Analysis

We started our analysis by assessing the proportion of confirmed HIV positive results following an HIV-ST screening test disaggregated by age-group, sex and district. We also assessed the changes in proportion of reactive HIV-ST results by month since the start of implementation in October 2018 until March 2020, as well as the monthly contribution of HIV-ST to overall case identification within the ZHCT project catchment area. Variables were summarized as frequencies and percentages and Chi-square tests were used for assessing differences in HIV positivity rates by age-group, sex, and month for each test modality separately as well as comparing within the groups. SPSS was used for data analysis (IBM Corp. Released 2017. IBM SPSS Statistics for Windows, Version 25.0. Armonk, NY: IBM Corp).

### Ethical Review

A non-research determination was granted by the Medical Research Council of Zimbabwe (approval number MRCZ/E/159), the local institutional review board which reviews research involving human subjects in Zimbabwe. Analysis for this manuscript used aggregate data routinely collected and reported by the ZHCT project, with no personal identifying information. None of the authors accessed patient-level data. Consent with each individual person for testing was obtained following national guidelines during implementation. None of the data can be linked back to the clients. Validation of the data was through the district monitoring and evaluation officers of which none of them are co-authors.

### Role of Funding Source

The ZHCT project is funded by the United States Agency for International Development under Cooperative Agreement No. AID-613-A-00009 and implemented in collaboration with the Ministry of Health and Child Care Zimbabwe. The funder did not play a role in interpretation of the results.

## Results

**Figure 1:**
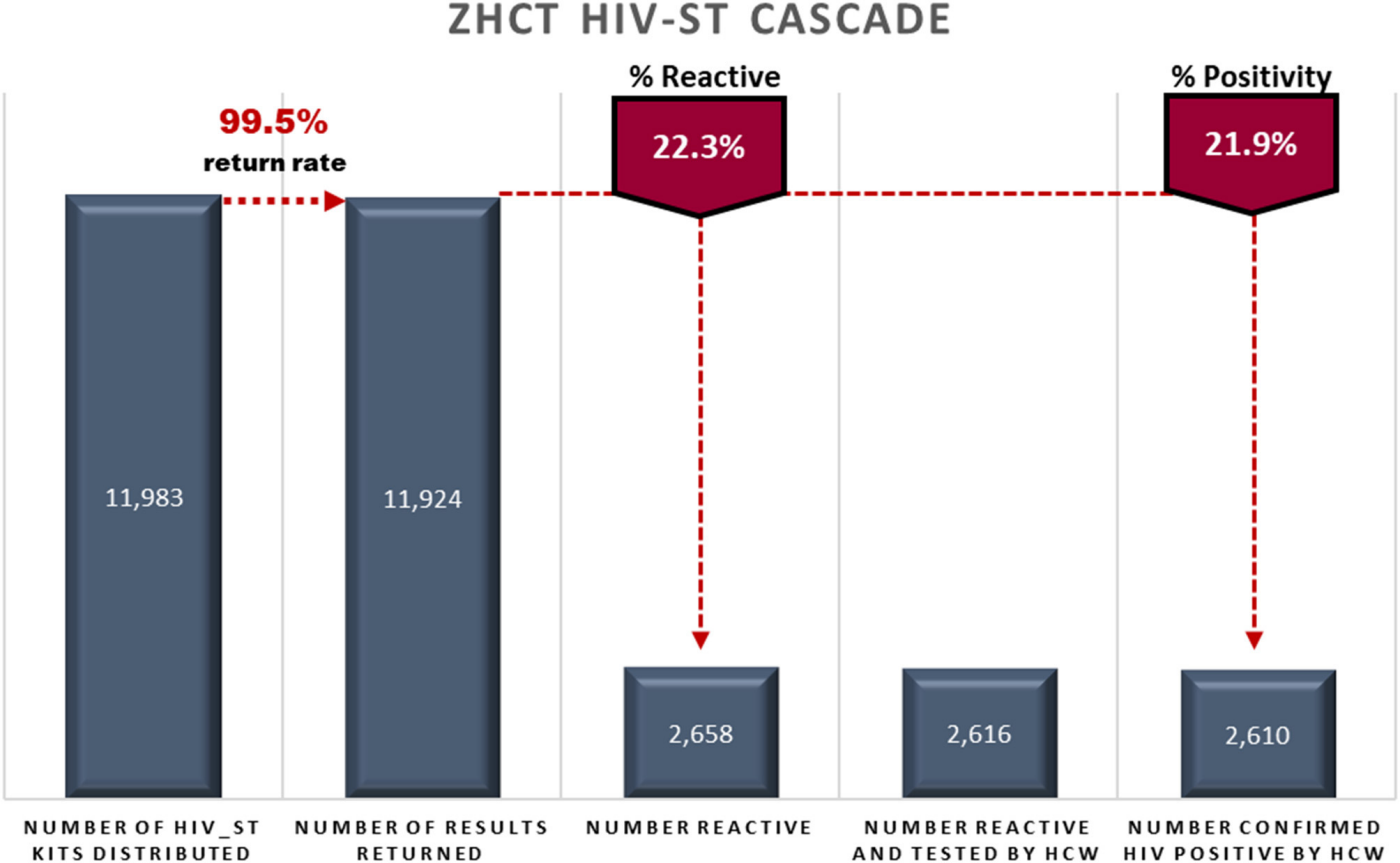
ZHCT HIV-ST cascade.

During the period October 2018–March 2020, the ZHCT project distributed a total of 11,983 HIV-ST kits, identifying 2,610 HIV positive individuals. Figure 1 shows the HIV-ST cascade for the ZHCT project for the period October 2018–March 2020.

Of the 11,983 HIV-ST kits distributed, 99.5% (11,924/11,983) were used and had results returned to the health care workers. Among those who returned HIV-ST results, 22.3% (2,658/11,924) were reactive and 2,610 were confirmed HIV positive by a trained health care worker using the national testing algorithm. This gave a concordance rate of 99.8% (2,610/2,658) between the HIV-ST positive results and the confirmatory testing an HIV positivity rate of 21.9% (2,610/11,924).

### ZHCT HIV-ST Positivity by Age, Sex, and District

Over a third (3,990) of clients who received HIV-ST were in the 35–49 age group. This age group also accounted for 39% (1,018/2,610) of the newly diagnosed HIV positive and had the highest positivity rate, 25.5% (1,018/3,990) compared to other age groups. Positivity rate varied across the different age groups, *p* < 0.001. More females (*n* = 6,422, 53.8%) accessed HIV-ST and had a significantly higher positivity rate (22.6%; 1/451/6,422) compared to males (21.1%; 1,159/5,502), *p* = 0.04. Table 1 shows the HIV positivity rate from the HIV-ST by age, sex, and district.

**Table 1:** Demographic characteristics of HIV-ST recipients and test results outcome.

	**Number of kits distributed**	**Number positive**	**Positivity**		***P*-value**
Age group			Positivity	95% CI	<0.001
<15 Yrs	48	3	6.3%	(0.60: 13.1)	
15–24 Yrs	2,536	419	16.5%	(15.8: 17.97)	
25–34 Yrs	3,840	918	23.9%	(22.56: 25.26)	
35–49 Yrs	3,990	1,018	25.5%	(24.16: 26.87)	
50+ Yrs	1,510	252	16.7%	(14.81: 18.57)	
Gender					<0.001
Female	6,422	1,451	22.6%	(21.6: 23.6)	
Male	5,502	1,159	21.1%	(20.0: 22.1)	
District					<0.001
Chivi District	582	207	35.5%	(31.6: 39.4)	
Gokwe South	2,507	424	16.9%	(15.5: 18.4)	
Gutu District	1,847	220	11.9%	(10.5: 13.4)	
Kwekwe District	2,422	713	29.5%	(27.6: 31.3)	
Makoni District	1,564	439	28.1%	(25.8: 30.3)	
Mutasa District	237	26	11.0%	(7.0: 15.0)	
Mwenezi District	2,549	489	19.2%	(17.7: 20.7)	
Zaka District	216	91	42.1%	(35.5: 48.7)	

When comparing districts, the highest positivity rate was reported in Zaka District (positivity 42.1%, 91/216) but more HIV positive individuals were identified in Kwekwe district (713/2,422), at a positivity rate of 29.5% during the study period. The difference in HIV positivity from HIV-ST varied across districts, *p* < 0.001.

### HIV-ST Positivity Over Time

**Figure 2:**
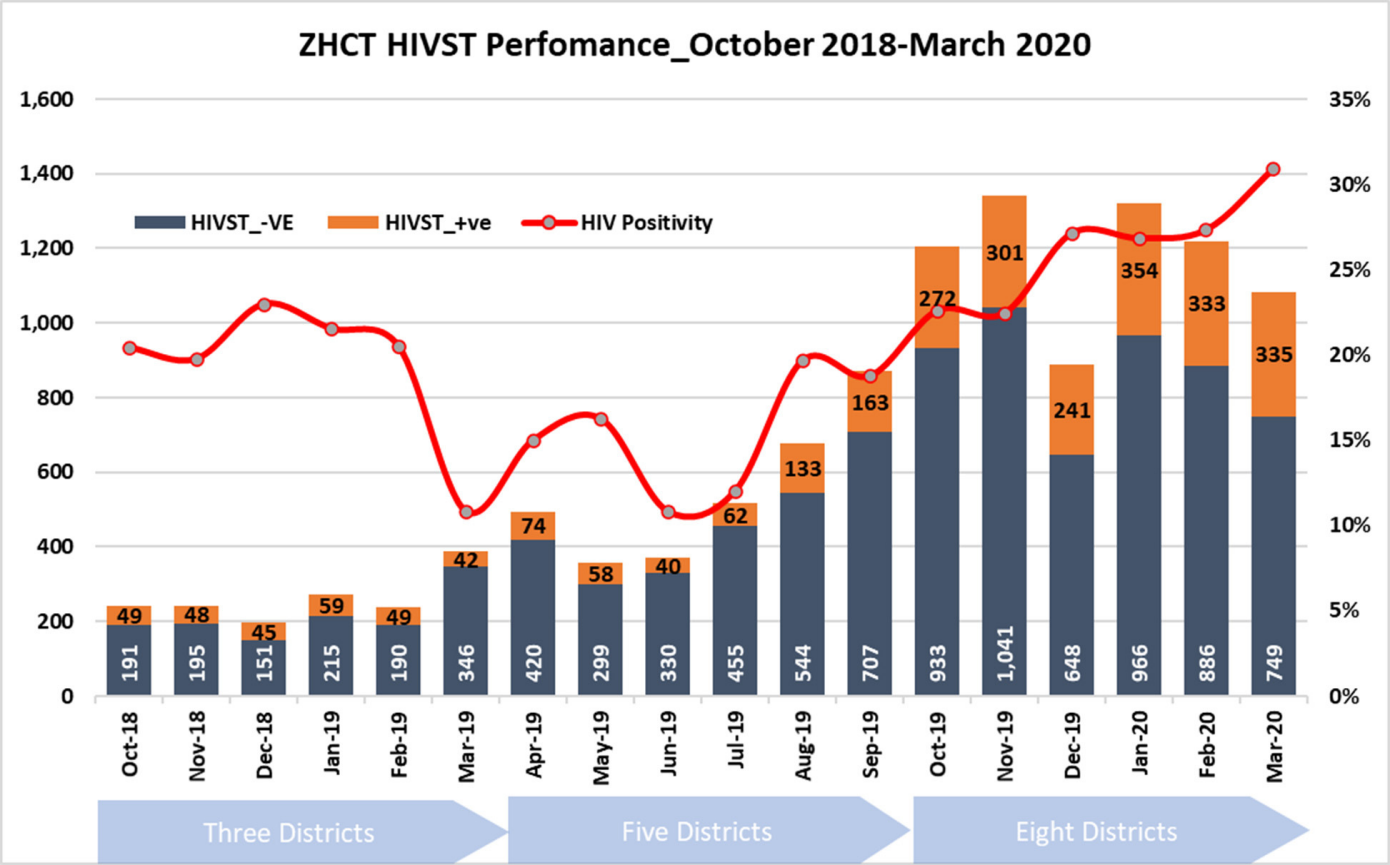
ZHCT monthly HIV-ST positivity rates: October 2018–March 2020.

The number of HIV-ST kits distributed gradually increased from an average of 200 kits distributed per month in the first 6 months, to more than 1,000 per month in November 2019. Figure 2 shows trends over time of HIV-ST test kits distributed and proportion which were reactive.

The proportion of reactive results averaged 20% in the first 6 months, declining in March 2019 before gradually increasing month-on-month from June 2019 to a high of 30% in March 2020.

### Contribution of HIV-ST to All Positives Within the Project

Overall, positivity rates from index and targeted mobile testing were higher at 34.7% (3,653/10,524) and in some age groups nearly double those of HIV-ST during the same period. This trend was the same for both age and sex. Table 2 shows the number of individuals tested and positivity rate for index and targeted mobile testing.

**Table 2:** Index and mobile testing results by age group, gender, and district.

	**Index and targeted mobile testing results**			
	**# HIV tested**	**# Positive**	**% Positive**	***P*-value**
Age-group				<0.001
<15 Yrs	2,740	270	9.85%	
15–24 Yrs	1,479	559	37.80%	
25–34 Yrs	3,060	1,225	40.03%	
35–49 Yrs	2,595	1,308	50.40%	
50+ Yrs	650	291	44.77%	
Total	10,524	3,653	34.70%	
Gender				<0.001
Male	5,577	2,092	37.50%	
Female	4,947	1,561	31.60%	
Total	10,524	3,653	34.70%	
District				<0.001
Chivi	973	322	33.08%	
Gokwe	2,250	752	33.43%	
Gutu	1,098	323	29.40%	
Kwekwe	2,887	1,113	38.54%	
Makoni	737	343	46.61%	
Mutasa	672	114	16.96%	
Mwenezi	1,043	443	42.47%	
Zaka	864	243	28.16%	
Total	10,524	3,653	34.70%	

Within each age-group, index testing and mobile testing were associated with higher HIV positivity rates compared to HIV-ST. This association was statistically significant for all age-groups except among those <15 years (Table 3).

**Table 3:** Differences in HIV positivity between HIV-ST and targeted/mobile testing.

	**Positivity rates by age-group**		**Test results**	
	**HIV-ST**	**Index/targeted mobile testing**	***X^**2**^***	***P***
<15	6.3%	9.9%	0.589	0.443
15–24 Yrs	16.5%	37.8%	134.43	<0.001
25–34 Yrs	23.9%	40.0%	107.708	<0.001
35–49 Yrs	25.5%	50.4%	197.696	<0.001
50+ Yrs	16.7%	44.8%	105.581	<0.001

**Figure 3:**
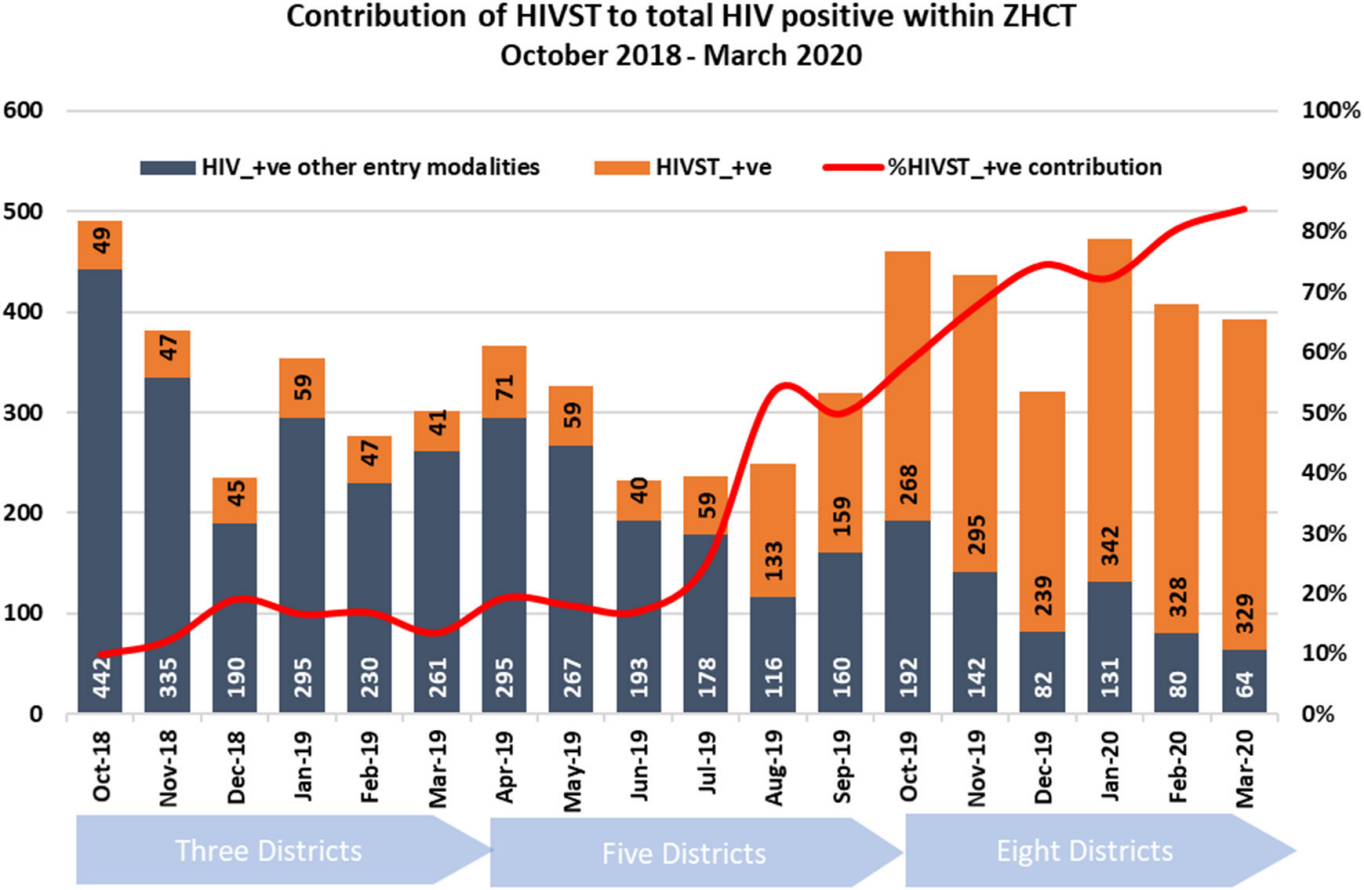
Monthly contribution of HIV-ST to total newly identified HIV positive within the ZHCT project.

Over time, the contribution of HIV-ST to HIV positives within the ZHCT project increased. Between October 2018 and July 2019, HIV-ST contributed around 25% of the total newly diagnosed cases within the project per month (Figure 3) and increased gradually, reaching 80% in March 2020.

## Discussion

The ZHCT project successfully scaled up HIV-ST and through this approach identified many HIV positive individuals who could have previously been undiagnosed. The high concordance rate of 99.8% between HIV-ST and confirmatory testing is reassuring and goes to show that HIV-ST is an accurate and effective strategy for HIV diagnosis. HIV-ST enabled over 2,600 individuals to know their HIV positive status. These findings are important for Zimbabwe and other countries that are close to reaching or have exceeded the 1st 95, where testing approaches need to be nuanced and better targeted. For these countries, HIV-ST should be scaled-up. In Zimbabwe, a gradual implementation allowed the project teams to learn and establish systems to monitor implementation before scale-up to other districts. By so doing, HIV-ST was targeted better and as result, HIV positivity improved month-on-month, and contributed more by month and over time to the overall number of newly diagnosed HIV positive individuals within the project. HIV-ST would reduce the workload for HCW, allowing them to spend more time on those who need care.

Multi-country evidence confirms high feasibility, acceptability and accuracy of HIV-ST across many delivery approaches, venues and populations, with minimal risk of harm (6–8, 14). Evidence on the effectiveness of HIV-ST during increased testing coverage is strong, while evidence on demand generation for follow-on HIV prevention and treatment services and cost-effective delivery is emerging. Despite these developments, HIV-ST delivery remains limited outside of pilot implementation (15).

This analysis from the ZHCT project, using routinely collected data has demonstrated how HIV-ST can be optimized at project level, generating lessons over time and targeting it to high-risk individuals. Over time, HIV positivity increased as did its overall contribution to total positives identified. Although the positivity from HIV-ST was lower than from index and targeted community testing in our program, it was at par or similar to that reported in other approaches (16). We attributed the lower positivity to the fact that HIV-ST was also availed to people who requested a test unlike nurse-tester conducted index and targeted mobile testing which is restricted to those who have been exposed to an HIV positive individual or have been screened to be high risk individuals. With 77% of all PLHIV already diagnosed in Zimbabwe (17), HIV-ST is an attractive testing modality to reach undiagnosed individuals.

A major concern for HIV-ST is the major drop off between test kit distribution, return of used self-test kits and confirmatory HIV testing (18). We achieved a very high return rate of 99.5%, together with the high concordance HIV-ST rates with confirmatory testing (99.8%). We attribute this to our implementation approach that was phased and closely supervised. We were able to integrate HIV-ST as part of index testing and use of symptom and behavioral risk prescreening. By offering confirmatory testing at home, we reduced the drop off from reactive tests to confirmation. Our success shows that HIV-ST can replace or complement less efficient testing strategies such as untargeted provider-initiated and community testing.

Our findings add to the body of evidence supporting the benefits of HIV-ST in general and how it has been used successfully to reach undertested populations. The potential of HIV-ST to increase access to and uptake of HIV testing has been highlighted (19) within antenatal clinic (ANC) platforms offering a unique opportunity to increase HIV couple testing among men (14). In Kenya, HIV-ST offered at ANC increased male partner testing by twelve times (20). In South Africa, when given a choice between clinic-based HIV testing and HIV oral self-testing, the overwhelming majority of young women chose HIV-ST offering an important opportunity to significantly increase testing rates among young women, their peers and partners (21). Males are known to have the highest HIV case identification gap worldwide while adolescent girls and young women (AGYW) also account for the majority of new infections. These pilot results in Kenya and South Africa, supported by performance of HIV-ST at the program level within the ZHCT project provides more evidence to support HIV-ST as an approach to finding men and AGYW, two demographic groups left behind. The utility of HIV-ST has also been demonstrated in Malawi where facility-based HIV-ST increased HIV testing among outpatients with minimal risk of adverse events (22). HIV self-testing was easily integrated into routine outpatient services and drastically reduced provider workload related to HIV testing while increasing testing coverage, including coverage among high-risk and hard-to-reach individuals (22). While in the Malawi study data were from a randomized control trial, our results provide a real-world view.

This study had some limitations. Firstly, implementation was stopped for 3 months at the start of COVID-19. We missed an opportunity to further scale-up and collect data which would have demonstrated the utility of HIV-ST during COVID-19 or similar emergencies. Secondly, we did not test any individuals who screened non-reactive on HIV-ST to determine if there were any false negatives under field conditions. However, OraQuick® HIV self-test kits have been shown to be very accurate with a specificity of 99.9% and a specificity of 93% (23). Additionally, because HIV-ST kits were only given to individuals who were identified through prescreening, we may have missed people who despite being at low risk may be HIV positive. Despite these limitations, these results offer important implementation lessons regarding HIV-ST. Lastly, the project only started disaggregating its data on distribution of HIV-ST kits by modality (home delivery vs. hotspot delivery) in November 2019 and we were not able to integrate that level of analysis in this study.

Our findings on the contribution of HIV case finding using HIV-ST have several implications during this COVID-19 period where major disruptions to healthcare delivery have occurred and health systems have become overwhelmed. Implementation of physical distancing measures and movement restrictions has further reduced access to health services (24) while the mounting fear of COVID-19 has also led to delayed health seeking (25). The WHO, UNAIDS, and the Global Network of People Living With HIV have worked collectively with national health departments and other development partners to ensure continued provision of HIV prevention, testing, and treatment services with particular efforts made to safeguard timely access to, and to avoid disruption of, routine HIV services (26). The priority, has been to ensure that PLHIV on ART continue to get their refills during the COVID-19 pandemic (27) HIV testing services, especially those requiring a blood draw and physical contact have been severely impacted. HIV-ST could offer an alternative that safeguards both clients needing to know their status and HCWs who administer tests especially during the COVID 19 pandemic when health facilities are closed, and health care workers are diverted to more critical roles. HIV-ST allows delivery of HTS while maintaining physical distancing between the patient and the HCW, be it at the healthcare facility or community level. In this context, delivery of test kits could be made even more safer by screening individuals for exposure or symptoms by phone, providing community workers distributing the kits with personal protective equipment, giving every newly diagnosed individual HIV-ST kits to distribute to their sexual contacts together with information on COVID-19 mitigation.

## Conclusion

FHI 360, through the ZHCT project has successfully implemented and scaled-up HIV-ST, achieving high return and positivity rates among those tested. These results demonstrate the potential of HIV-ST to supplement other testing modalities toward the achievement of the UNAIDS 95-95-95 goal. HIV-ST should be scaled-up to ensure that the trajectory for countries to reach epidemic control does not stall.

## Data Availability Statement

The data analyzed in this study is subject to the following licenses/restrictions: The data sets are available on request from the project. Requests to access these datasets should be directed to Auxilia Muchedzi, amuchedzi@fhi360.org.

## Ethics Statement

Ethics approval was not required for this study, as according to United States federal guidelines and guidance from the FHI 360's Office of International Research Ethics, this analysis did not constitute human-subjects research. Additionally, a non-research determination was granted by the Medical Research Council of Zimbabwe (approval number MRCZ/E/159), the local institutional review board which reviews research involving human subjects. Written informed consent from the participants was not required to participate in this study in accordance with the national legislation and the institutional requirements.

## Author Contributions

AM, MM, and TTaf conceived and designed the manuscript. FM, TM, HS, RD, TZ, TTap, TS, TN, and GN wrote the manuscript. MB participated in the revision of the manuscript. All authors made substantial contributions to conception and design, acquisition of data, or analysis and interpretation of data, critically revised manuscript and approved the final version.

## Conflict of Interest

The authors declare that the research was conducted in the absence of any commercial or financial relationships that could be construed as a potential conflict of interest.
